# Popliteal Artery Entrapment Syndrome (PAES) in a 17-Year-Old Adolescent

**DOI:** 10.1155/2019/8540631

**Published:** 2019-03-11

**Authors:** Viktor Labmayr, Aryan Aliabadi, Kurt Tiesenhausen, Marianne Brodmann, Florian Schmid, Dana Moore

**Affiliations:** ^1^Department of Orthopaedics and Trauma, University Hospital Graz, Austria; ^2^Division of Angiology, Department of Internal Medicine, University Hospital Graz, Austria; ^3^Division of Vascular Surgery, Department of Surgery, University Hospital Graz, Austria; ^4^Division of Neuroradiology, Vascular and Interventional Radiology, Department of Radiology, University Hospital Graz, Austria

## Abstract

**Introduction:**

Popliteal artery entrapment syndrome (PAES) is caused by compression of the popliteal artery (PA) due to deranged myotendinous structures. It can be asymptomatic or may present with exercise intolerance, claudication, or even limb-threatening ischemia. The clinical picture depends on the anatomy and degree of vascular compromise.

**Case Description:**

We report a case of a 17-year-old Caucasian male with PAES Type II presenting with intermittent claudication and progression towards acute limb ischemia.

**Diagnostics:**

MRI and MRA helped identifying the aberrant anatomy and thrombotic occlusion. Doppler ultrasound and conventional angiography have also been employed in a stepwise approach.

**Intervention:**

The thrombus at the site of occlusion was removed by the use of catheter-directed lysis. Subsequently, popliteal artery release was achieved by myotomy of the aberrant medial head of gastrocnemius muscle (MHGM) and muscle transfer to the medial femoral condyle. A three-month regimen of 60mg edoxaban was recommended after surgery.

**Outcome:**

Surgical correction of the anomalous anatomy and postoperative anticoagulation led to freedom of symptoms.

**Lesson:**

Clinical presentation of PAES mimicking peripheral artery occlusive disease is very rare but potentially limb-threatening. PAES should be considered in young and otherwise healthy individuals.

## 1. Introduction

Popliteal artery entrapment syndrome (PAES) is a very rare pathology caused by an abnormal anatomic course of the popliteal artery (PA) and/or the adjacent myotendinous structures of the popliteal region. The clinical picture is highly variable. PAES can be asymptomatic in the case of sufficient collateralization of the PA. However, it can also present with foot paleness, pulselessness, and intermittent or even constant claudication. Its most critical manifestation is acute limb-threatening ischemia. The degree of symptoms depends very much on the underlying morphology. Functional entrapment caused by hyperemic muscle is neither visible in clinical examination nor detectable in Doppler ultrasound study; hence detailed medical history-taking can give the clue for its diagnosis. Partial or complete obstruction of the PA can be initially assessed by taking the pulses or by visualization with ultrasound.

## 2. Case

We report the case of a previously healthy 17-year-old Caucasian male with exercise-induced pain of the right foot that had deteriorated over the course of three months. During the last week prior to presentation at our emergency department, the symptoms had progressed from intermittent to constant calf pain. At clinical examination, the right foot was pale and cool with delayed capillary refill and absent pedal pulses consistent with the clinical picture of acute limb ischemia. The left foot was warm and well perfused with palpable pedal pulses.

Doppler ultrasound study of the right lower limb showed an occlusion at the proximal part of the right popliteal artery (PA). With magnetic resonance angiography the presence of a four-centimeter thrombus occluding this section of the PA was confirmed.

Furthermore, Doppler ultrasound study of the asymptomatic, contralateral (left) popliteal region revealed an occlusion of the PA with sufficient collateralization and unimpaired three-vessel runoff to the foot.

**Figure 1:**
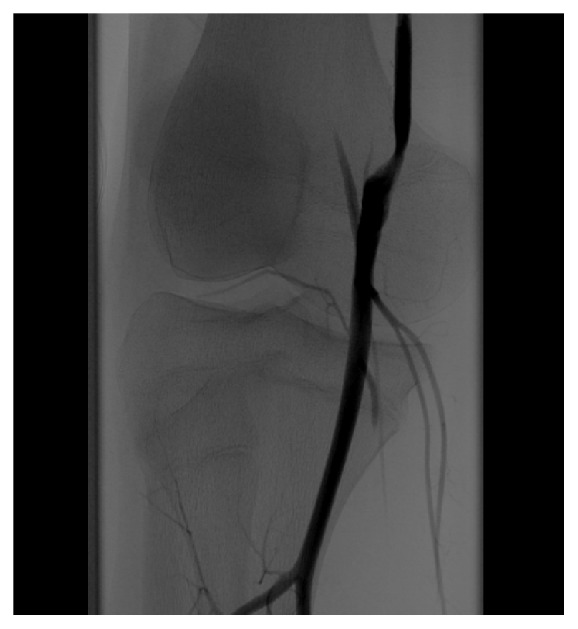
Digital subtraction angiography (DSA) of the PA with occlusion and hourglass configuration suggesting extravascular causes for lumen narrowing.

By conventional transfemoral angiography, the previously identified occlusion of the right PA and its aberrant course were demonstrated again (Figure 1). An attempt of continuous intraarterial thrombolysis of the occluded right PA was undertaken by the use of actilyse and continuous heparin applied via a lysis catheter over 24 hours. The required therapeutic dose was controlled with aPTT between 60 and 80 seconds. This resulted in a significant reduction of thrombotic material within the PA, but the vessel was still considerably occluded (70-80%). After 24 hours of catheter-directed thrombolysis, low-molecular-weight heparin (LMWH) in therapeutic dosage and continuous infusion of iloprost (over 4h) were given daily. Further investigations were started to identify the already suspected extravascular cause of occlusion.

**Figure 2:**
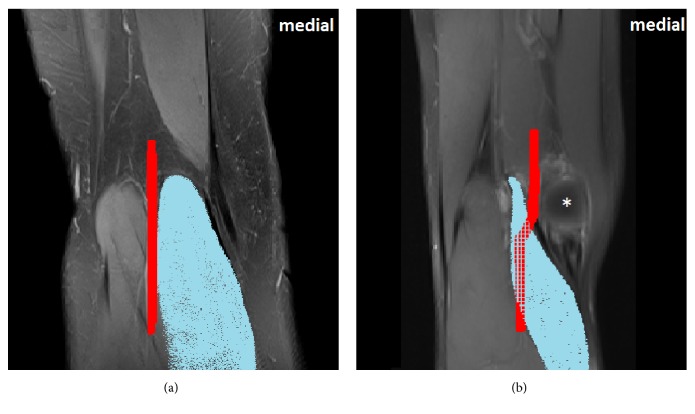
MRI of the right knee with regular anatomy (a, left) in comparison to our patient with PAES Type II (b, right). In the right image, the MHGM (light blue) is lateralized from its regular origin at the medial condyle of the femur (asterisk) and compresses the medially displaced PA (red).

Indeed, in magnetic resonance imaging the anomalous origin of the MHGM lateral to the medial condyle of the femur was detected. The muscle's tendon and belly crossed over a medialized PA and caused its compression (Figure 2(b)). This aberrant anatomy was present bilaterally, yet asymptomatic on the left side.

Hence, indication for surgical decompression was given. A posterior surgical approach was used to enter the popliteal fossa. The intraoperative anatomy presented as a PAES classification Type II with a popliteal artery course medial to the extremely lateralized MHGM. The artery did not show signs of stenosis or vascular wall hypertrophy; therefore only myotomy at the origin of the MHGM was performed. The artery was moved laterally into the popliteal fossa and the muscle was inserted at the correct position at the posterior surface of the medial femoral condyle.

Postoperatively, the patient recovered well and had a warm, pain-free foot with palpable pedal pulses. A three-month regimen of 60mg edoxaban was recommended after surgery.

## 3. Discussion

Patients under 40 years of age presenting with arterial occlusive disease of the lower limbs may suffer from a rare disease. Although premature atherosclerosis is the most common problem, claudication with limb-threatening ischemia may result from thromboembolism, Buerger's disease, collagen vascular disease (both autoimmune or hereditary), popliteal artery aneurysm, adventitial cystic disease, and popliteal artery entrapment syndrome [1–5]. Our patient had a negative history of risk factors associated with arterial occlusive disease and normal laboratory values regarding inflammation or autoimmune disease. Hence, local lumen narrowing due to macroscopic structural changes was highly suspected. In this setting, MRI is key to identifying the culprit. On imaging, the abnormal relationship between the PA and the surrounding myofascial structures of the popliteal fossa gave the hint for diagnosing PAES.

The syndrome can be classified into six subtypes based on the anatomic abnormality. In Type I, the PA runs medial to the normally positioned MHGM. In Type II, the MHGM attaches abnormally more laterally on the medial femoral condyle and medially displaces the PA. In Type III, an abnormal accessory slip arises from the MHGM and entraps the anatomically normal PA. In Type IV, the PA lies deep to the popliteus muscle that causes its compressions. Type V involves both the popliteal artery and vein in any of the before mentioned scenarios. Type IV describes a functional entrapment of the PA caused by an anatomically regular but hypertrophied MHMG [6–8]. Although not included in this classification, entrapment syndrome may also arise from anomalous origin of the lateral head of the gastrocnemius muscle [9].

This case report is about a patient with PAES Type II that became symptomatic on the right limb and remained asymptomatic on the left limb. Studies report the proportion of bilateral entrapment of the PA ranging between 30 and 74 percent [8, 10, 11], highly emphasizing the need for bilateral diagnostic work-up.

Although intraarterial lysis brought significant reduction of thrombotic occlusion, surgical release of the entrapped PA was necessary. Intraoperatively, the integrity of our patient's PA could be verified by surgical exploration. Theoretically, constant mechanical compression of the artery by myotendinous structures might have induced sclerosis and aneurysmal degeneration of the arterial wall resulting in stenosis, aneurysm, or occlusion. Given this scenario, further vascular procedures such as venous graft reconstruction, endarterectomy, or bypass grafting would have been necessary [12–14]. In this patient, myotomy with restoration of the regular anatomy was sufficient to result in cure and freedom from symptoms in the postoperative course.

We recommended oral anticoagulation to decrease the thrombogenic risk after occlusion caused by entrapment. Hypothetically speaking, compression from outside causes blood flow deceleration and stasis, increasing the risk for thrombotic events. We did not recommend antiplatelet therapy as there is assumedly no endothelial dysfunction in artery entrapment syndrome cascading platelet aggregation.

## 4. Conclusion

PAES is rare, but knowledge and physicians' awareness of this condition are crucial to early diagnosis and treatment in cases with possibly limb-threatening claudication. The pathology may affect otherwise young and healthy individuals without cardiovascular risk factors. Interdisciplinary cooperation between several specialists such as angiologists, radiologists, and vascular surgeons is fundamental for optimal patient management.
